# Psychiatry

**Published:** 1907-12

**Authors:** F. St. John Bullen


### PSYCHIATRY.

**Bacterial origin of general paresis.**—Few investigations into the pathology of insanity have been followed with more interest than those of Ford Robertson and McRae[2], dealing with a bacterial origin for general paralysis of the insane. These authors assert that a diphtheroid bacillus—perhaps an attenuated form of Klebs-Löffler bacillus, but more likely a distinct form—is the specific organism in general paresis and tabes. The invasion of this is specially predisposed to by syphilis, chronic alcoholic excess, and over-nitrogenous ingestion. Large numbers of a diphtheroid bacillus are said to have been found in the respiratory, alimentary and genito-urinary tracts of advancing cases of general paresis, the principal infecting sites being considered to be the buccal and naso-pharyngeal mucosæ. From these foci toxines are generated which occasion the facial tremors and paresis, and by perineural conduction some of the cortical lesions also. Stress is laid on the actual tissue-invasion by these organisms in general paresis as distinct from their finding on the surfaces of mucosæ, which may happen in other cases than general paresis or in sane individuals. The authors describe especially two forms of diphtheroid organism, one often a thread-like bacillus which they term B. paralyticus longus, commonly the only micro-organism found in the catarrhal pneumonic foci that occur, in most of the cases of general paresis dying during congestive seizures. A second form of diphtheroid bacillus, which resembles the B. xerosis in broth reaction, but differs morphologically in showing prominent metachromatic granules, is not thread-like, and is thinner and shorter than the other; this they call B. paralyticus brevis. Both forms are often virulent to mice and rats, but there is a variance in their relative toxicity. The B. paralyticus brevis has been detected in fresh blood in one case of general paresis, but is said to be taken up so rapidly by the polymorphonuclear leucocytes, that complete digestion occurs in two to three hours. This bacteriolytic power of the leucocytes in general paresis, the authors think, shows a higher index than in the normal individual, and it is owing to this, in

[1] *Loc. cit.* [2] *J. Mant. Sc.*, 1907, liii. 590.

the greater number of cases, that it is not practicable by present methods to procure cultures during life. They, however, made pure growths of a diphtheroid bacillus from fresh blood in four cases and from cerebro-spinal fluid in two. Disintegrating bacilli were found in the blood, cerebro-spinal fluid, urine, vessel-walls and pia-arachnoid of general paresis in many cases. Their experiments on rats gave varying results according to the inoculation with the long or short form of the diphtheroid, and the isolation of these from dead or living tissues. Those rats which died with paralytic symptoms presented changes in their cerebral tissues in the form of periarteritis, neuroglia-proliferation and nerve-cell lesions. Robertson and McRae believe there are two varieties of general paresis similar and yet essentially distinct, occasioned by one or other of these bacilli, or perhaps by yet another form, or forms, of diphtheroid bacilli, gaining entrance to the lymph stream and blood. They lay stress on the benefit found in a case of tabetic crises, in whom injections were at first made of definite doses of a killed culture of the B. paralyticus brevis (isolated from the patient's urine), and subsequently of a bactericidal serum. The first injections were followed by the return of the crises, which seemed to be immediately dependent on them. The aim in these researches being a therapeutical one, experiments were commenced with an anti-bacterial serum, prepared by inoculating sheep with dead cultures of the B. paralyticus longus. The immunised serum was given either by hypodermic injection, by mouth, nose, or in one case per rectum. Previous inoculations with vaccines, prepared by suspension of the bacilli in sterile saline solution, gave encouraging results. Thirty-four cases of general paresis were treated with anti-sera, and in all what was considered a diagnostic reaction for general paresis and tabes obtained. This consisted in a rise of temperature to 100° F. or more (or 99° F. or more when given by mouth), occurring within twelve and over within twenty-four hours in the case of injections. Besides this there were flushings, vertigo, defect of vision, confusion, increased inco-ordination, &c. Control experiments were made in other forms of insanity, and in these no specific reaction was observed after mouth administration, and any rise of temperature after injection was explicable by other factors. (This qualification hardly appears satisfactory to us.) Leucocytic increase after the anti-sera was found in most cases of these controls, but there was no material change in the paretics. On the other hand, normal serum and polyvalent anti-streptococcal sera were injected into general paretics without reaction. The authors claim that most of their cases of general paresis treated with the sera undergo more or less improvement; thus, of 12 cases treated by themselves all improved; of 9 cases treated by others 6 improved, 3 did not. This leaves 13 cases unaccounted for.

George Robertson, of Stirling, has carried on investigations with the same objective, but with yet more elaborate precautions and technique. In thirteen general paretics he found present a constant diphtheroid organism in seven, and he obtained cultures from the blood in seven cases and from cerebro-spinal fluid in four cases.

Thus, not only were cultures more frequently obtained than by Ford Robertson and McRae, but the organism was studied in more than twenty cultural media. Moreover, the bacillus described by the latter authors had not been observed by him in any case, and the organism which he had found differed from it in many respects. It also had been found in other forms of insanity in acute cases. G. Robertson thinks it possible that a large group of diphtheroid bacilli may exist which produce substances toxic to the nervous system, and which, if not specific in producing general paresis, may be contributory.

Orr and Rows hold that infection in general paresis, bacterial or toxic, proceeds along the lymph paths rather than the blood stream. Candler, working at the Claybury laboratory, quotes various bacteriologists as showing that diphtheroid organisms, in the first place, are met with all over the body in cases free from insanity; further, that organisms similar to those described by Ford Robertson are met with in two localities, especially mentioned by this author, in a large number of sane persons; that diphtheroid organisms are, as a whole, met with in no greater frequency in general paretics than in other forms of insanity, and cannot be isolated more readily from the one than from the other.

Candler has examined the blood of twenty-four general paretics during life — on forty-one occasions in all, and in no single instance has he seen or obtained cultures of an undoubted diphtheroid bacillus. Also, in nine cases in which he has examined the cerebro-spinal fluid, he has neither seen diphtheroids nor obtained cultures. He comments on the dangers of contamination during culture making, the doubtful evidence of stained films and sections unless confirmed by cultures, and the errors caused by terminal and *post-mortem* invasions

Lewis Bruce has found **hyperleucocytosis** in what he terms **bacterial toxic insanities,** and also, variably, in **general paresis.** This increase, together with certain temperature rises—regular, recurrent or irregular in type according to the stage of the disease —with disturbances of alimentary tract, certain serum agglutinations, with signs of a general toxic invasion and lowered leucocytic resistance, leads him to consider general paresis as a disease of bacterial origin. He has found, in three or four cases, serum taken from general paretics in a stage of remission to arrest the progress of early paretics. One patient he immunised to the Ford Robertson diphtheroid, and completely stayed the course of the disease; death occurred ere long from phthisis. Bruce recognises, however, that severe attacks of erysipelas, carbuncle, &c., may also indefinitely cause an arrest.

It will be seen that so far there is not much unanimity amongst different observers as to the existence or determination of the bacterial organism in general paresis. Nevertheless, undoubted progress is being made in an investigation of immense difficulty, and the results of Ford Robertson's serum treatment cannot but be viewed with hopeful bias. That such advance has been made since 1894, when an article by Goodall and Bullen reviewed the then meagre literature, and to some extent forecast the present attitude towards general paresis, is productive of a reasonable expectation that the further researches of Robertson and others may end in a solution of a most involved question.

Pomeroy[1] has contributed very interesting and important evidence as to the value of **lumbar puncture in the diagnosis of syphilis, general paralysis** and some other conditions. The presence or absence of leucocytic and albumin increase, separate or conjoined, in the cerebro-spinal fluid appears to give much help, under known conditions, in the determination of these diseases. The number of experiments collected and personally made by Pomeroy are sufficiently harmonious in result to make his conclusions of considerable weight. Leucocytic increase seems almost the rule in secondary syphilis; in the tertiary form not so surely, unless the nervous system be involved. The amount of increase is, however, seldom so great in syphilis as in general paresis, nor is the albumin content, even if at all present, in the former nearly so abundant as in the latter. The estimation of albumin is considered a most important matter in deciding on the presence of general paresis, and is in many cases the only feature that will differentiate it from syphilis. Albumin increase is, however, not so constant as hyperleucocytosis, nor is it always parallel with it. The whole clinical picture of a doubtful case must, of course, be carefully studied; but in such, where the poise of opinion is between brain syphilis and general paresis, a relatively small cell increase and low albumin content may justify decision for the former. On the other hand, a negative finding of leucocytic and albumin increase is of more value in deciding against syphilis and general paresis than a positive one is for them.

Hyperleucocytosis in general paresis has been observed in nearly all of 500 cases, lumbar punctures giving negative results of only 2.6 per cent., after due allowances have been made. Pomeroy himself in thirty clear cases of general paresis found

[1] *J. Nerv. & Ment. Dis.*, 1907, xxxiv. 312.

positive increase in cell and albumin in every case. (In about half of these he believes syphilis to have been present.) His opinion is that these conjoined positives constitute one of the earliest and most constant signs of general paresis, even appearing before amnesic, eye or ataxic symptoms.

Farrar regards the hyperleucocytosis as the outcome of a subacute or chronic cerebro-spinal periarteritis or pia-arachnitis. Further experiments have been made to ascertain the value of this cell and albumin content in the differentiation of chronic alcoholic psychoses and other central organic brain troubles. Nissl collected and examined thirty cases of presumed chronic alcoholic psychoses; in twenty-three of these the finding was negative; the others proved to be either actual or probable paretics or old syphilitics. Other observers have recorded similar results. The finding in epilepsies is usually negative, except where there is a syphilitic basis; the same is said of brain tumour, arterio-sclerotic insanity, Korsakoff's disease, central hemorrhages, dementia precox, and paranoidal excitement. In short, a constant absence of hyperleucocytosis practically negatives brain syphilis and para-syphilitic conditions. On the other hand, the differentiation of brain syphilis and general paresis will have to rest on the relative amount of hyperleucocytosis and albumin association.

Pomeroy insists on the necessity of several punctures before decision be made. The dangers of the operation appear minimal if not more than 3 to 5 c.c. of fluid are withdrawn; it is, however, counterindicated in cerebellar tumour. The patient should not be punctured unless put to bed; in this case mild headache, nausea or vomiting seem the only sequelæ.

**Change in type of general paresis.**—Clark and Attwood[1] have collected 3,000 records of general paresis, to investigate the question of a change in its type. They assert that the oft-stated opinion that there is a relative increase of the simple demented and melancholic type to the grandiose in late years is mainly due to the inclusion of cases of dementia, depression, and cerebral syphilis, formerly wrongly classified, but now correctly grouped under general paresis. These authors consider euphoria still to be the characteristic psychical symptom, and to be found in 70 per cent. of the cases, although less extravagant and grotesque. Allowing for the more rigid delimitation of pseudo-general paretics, the writer of this periscope has seen no reason to alter the conclusions expressed by him in 1893 on this variation in type—at any rate, so far as England is concerned. He still asserts that mania is relatively less frequent, less pure and less sthenic; that primary dementia is more common; convulsive

[1] *J. Nerv. & Ment. Dis.*, 1907, xxxv. 553.

and paretic seizures less frequent, less violent and fatal. The variations are much influenced by locality, and the strenuous life and fresh population of the American cities quite accounts for a maintenance of the former characteristic type there. It is likely, on the other hand, that any gradual process of degeneration taking place in a nation is reflected in the type of its insane as well as its sane population.

F. St. John Bullen.

